# The health of workers in the global gig economy

**DOI:** 10.1186/s12992-018-0444-8

**Published:** 2018-12-18

**Authors:** Uttam Bajwa, Denise Gastaldo, Erica Di Ruggiero, Lilian Knorr

**Affiliations:** 1 Dalla Lana School of Public Health, University of Toronto, 155 College Street, Office 409, Toronto, ON M5T 3M7 Canada; 2 Lawrence S Bloomberg Faculty of Nursing, University of Toronto, Toronto, Canada; 3 Royal Bank of Canada, Toronto, Canada

**Keywords:** Work and health, Mental health, Surveillance, Globalization, Precarity, Workers’ rights

## Abstract

**Background:**

The “gig” economy connects consumers with contractors (or workers) through online platform businesses to perform tasks (or “gigs”). This innovation in technology provides businesses and consumers access to low-cost, on-demand labour, but gig workers’ experiences are more complex. They have access to very flexible, potentially autonomous work, but also deal with challenges caused by the nature of the work, its precariousness, and their relationships with the platform businesses. Workers in the Global North and South may also experience these challenges very differently. Based on our report “Towards an Understanding of Canadian Workers in the Global Gig Economy”, we present a commentary on the implications of a globalized online platform labour market on the health of gig workers in Canada and globally.

**Main body:**

Based on our scoping review of peer and grey literature, we categorized gig worker vulnerabilities in three ways: 1) occupational vulnerabilities, 2) precarity, and 3) platform-based vulnerabilities. Occupational vulnerabilities are connected to the work being performed (e.g. driving a car or computer work) and are not specific to platform labour. Precarity refers to the short-term, contingent nature of the work, characteristics that may be shared with other forms of work. Some examples of precariousness are lack of health insurance, collective bargaining, or career training and promotion. Finally, platform-based vulnerabilities are particular to the way platform labour is structured. These vulnerabilities include worker misclassification, information asymmetries, and the culture of surveillance. We suggest that, together, these vulnerabilities challenge gig workers’ right to health.

**Conclusions:**

We propose that the experience of gig workers around the world must be understood in the context of neoliberalism, which has increased both the globalization and precaritization of work. While gig workers share some vulnerabilities, which have important negative consequences on their health, with other workers, the platform-specific vulnerabilities of workers require further inquiry. In particular, the specific health and overall experience of gig workers in different regions of the world – with different labour policies and sociopolitical contexts for work – must be disentangled as workers in the Global North and South experience this work very differently.

## Background

Developments in online technology have made it possible for people and businesses around the world to participate in a growing, global marketplace for contract labour. Consumers and companies can use digital platform businesses to contract out manual tasks, transportation, and “human intelligence tasks,” such as tagging photos or transcribing a podcast. Businesses like Uber, TaskRabbit, Amazon Mechanical Turk, and many others exist to lower transaction costs by connecting consumers to contractors, on demand. These platforms have facilitated the disaggregation of work into smaller and smaller units, sometimes called “micro tasks”, a trend known as the “unbundling of work” [1]. Platform mediated work can be done locally for tasks that require human contact (driving, caregiving) or anywhere in the world, in the case of online tasks (transcriptions, graphic design). Because of these innovations, “gigs” – short-term, one-off employment contracts mediated by platform businesses – have the potential to transform the future of work globally. Our current understanding of the social and health effects of this emerging labour economy is limited. This commentary elaborates on some of the findings from a scoping review we conducted to understand workers’ experience of online platform mediated labour [2].

## The effects of gig work on workers

In our report “Towards an Understanding of Canadian Workers in the Global Gig Economy” (2018), we present findings from a scoping review of both peer and grey literature on Canadians participating in platform labour markets, with particular attention to the health effects of gig work**.** We found that in spite of the growth in the gig economy – and widespread media coverage of specific platform businesses like Uber and TaskRabbit – very little is known about workers’ characteristics, motivations, experiences, and the health consequences of their work.

Certain vulnerable groups appear to be overrepresented in the gig economy, for instance young people (millennials), who experience greater unemployment rates in many countries, and people with lower incomes, who may already be working multiple jobs. Gig work is precarious, meaning it is often low paid, temporary, provides no training, health, or retirement benefits, and shifts more of the risk of doing business from the employer to the worker. Even though precarious work and working conditions generate and reproduce health inequities within and between countries [3], the platform labour economy has also generated opportunities for flexible work, entrepreneurship, and business innovation [4].

Accordingly, empirical studies about gig workers reveal this tension between necessity and opportunity, showing that gig work is experienced very differently between platforms and across regional and demographic lines [2]. For instance, workers living in Low and Middle Income Countries may have access to higher wages through online global labour markets than in their countries of residence [5]. Similarly, some groups, like students or caregivers, may be attracted to the flexibility and independence of gig work as it allows them to work around unpredictable schedules [4]. Nevertheless, we found workers share health and social vulnerabilities when participating in online business platforms, such as the lack of health insurance and social benefits. We have organized them in three categories described below. These vulnerabilities have implications for research, policy, and programs supporting workers.

### Three categories of health and social vulnerabilities

Based on our scoping review, gig workers’ health and social vulnerabilities can be divided into three overlapping categories: 1) occupational vulnerabilities, 2) precarity, and 3) platform-based vulnerabilities. While all three shape the experience of workers, we identified platform-based vulnerabilities as the biggest gap in understanding how this new form for global labour effects workers’ right to health.

#### Occupational vulnerabilities

The occupational vulnerabilities of gig work are specific to the type of work being performed and are shared with others doing similar work outside of platforms. These might be occupational health risks like an increased risk of traffic accidents for Uber drivers and bike couriers or musculoskeletal injuries associated with repetitive tasks like typing. They also include the potential danger of entering an unfamiliar home to provide cleaning or care-giving services. These challenges are made worse in jurisdictions without appropriate occupational health regulations and enforcement [6].

#### Precarity

Similarly, the health vulnerabilities related to the short-term, contingent nature of the work are analogous to those faced by many precarious workers and are a product of the erasure of the employer-employee relationship created by platform-mediated contracts [2]. Workers are vulnerable to the economic and social demands of providing their own tools and equipment, limited opportunities for training and career growth, low wages, no job or income security, and wage discrimination against certain groups, particularly women [6, 7]. Gig workers also share health risks associated with the psychological distress of precarious work and lack of health and social insurance coverage in countries without publicly funded health systems. The literature’s focus on the United States shows how the lack of comprehensive access to universal health coverage and social benefits compounds the deleterious effects of precarious work. Looking specifically at the online labour market (work that does not require providing services in-person), businesses can contract workers anywhere in the world, which creates a race to the bottom for lowest remuneration [3]. It also means that businesses may be contracting workers in countries where labour laws and access to health care are quite poor.

#### Platform-based vulnerabilities

Finally, gig workers’ health and well-being are affected by the way platform businesses are designed and operated, including issues like worker classification, control of pricing and workflow, social isolation, menial micro-tasks, and work-related stress due to surveillance.

Globally, the debate around whether gig workers are being misclassified as contractors, rather than employees, is a key issue [6]. This is related to precarity, because as independent contractors, gig workers lack stability and benefits associated with being an employee, but misclassification is also a platform-specific challenge because online businesses shape work relations in particular ways. For instance, algorithms created by the platform businesses determine pricing and access to work, which means workers are unable to negotiate prices (something contractors may be able to) or are required to agree to a price before knowing the full extent of the work.

Employee misclassification and lack of control over rates occur in the context of social isolation and the unbundling of work. Workers engage platforms as self-employed contractors, usually doing very independent work, and there are no mechanisms for connecting with other workers using the same platform. Platforms also create a disconnection between workers and the work itself because digital work is sometimes organized into “microtasks” that are tedious, short-term, and detached from a larger goal that might bring meaning to the work or promote a worker’s professional development. Behind technological services there are many completely anonymous workers, working multiple jobs in isolation, sometimes for platforms in different countries, and lacking social integration and a sense of belonging through an identified profession or form of employment.

Another feature of platform-based work is more insidious. Platform surveillance and evaluation of workers has considerable psychosocial effects [8]. Businesses monitoring workers through apps know when workers are logged in, their locations, and, rumours suggest, can even eavesdrop on interactions with customers. In addition, the rating systems used in virtually all platform businesses to establish trust between workers and clients are frequently described as a key source of worry for workers, who may feel punished and lose revenue for factors outside of their control. Factors might include bad traffic, in the case of Uber drivers, or customer racial or gender prejudices, in response to online profiles [9]. Low ratings mean workers can be “deactivated” (basically, fired) from a platform business with no recourse. As a result, workers feel pressured to perform emotional labour to please customers (being exceptionally affable, tolerating inappropriate behaviour from users), which can be mentally exhausting and stressful. Despite living under digital surveillance, workers (and researchers) do not have access to the big data that platform businesses generate, allowing for data to be asymmetrically used to exercise power over workers. Overall, the psychosocial effects of workers’ interactions with the platform are poorly understood, though their potential to harm workers, and their communities, is clear.

## Conclusions

Though neoliberalism has increased precarization internationally and deepened the income gap within and between countries [3, 7] – significantly affecting employment relations and health – gig workers’ experiences differ from those of other precarious workers. While gig workers share occupational vulnerabilities and features of precarity with other workers, such as income insecurity, they also confront platform-based vulnerabilities. We propose that further studies of the experience of workers in the global gig economy should disentangle the larger effects of neoliberalism from the specific features of the gig economy. The dialectics of individual choice/agency and market-shaped occupation/structural forces requires more research.

Given the complexity, global reach, and opacity of the gig economy, greater conceptual clarity and more empirical research are needed about the effects of gig work on workers’ health and experiences. Our review focussed on the experiences of Canadian workers and few empirical studies exist on gig workers in the Global South [2]. Nevertheless, we suggest that the placelessness of gig work demands sensitivity from researchers to the ways individuals interact with the global platform labour market and how distinct groups of workers residing in different regions experience that market.

The vast array of work opportunities and vulnerabilities in the gig economy make unified, worker-led responses challenging. While activist groups, unions, and consumers have demanded greater informational transparency around issues like product sources and production networks for many companies, in the gig economy users (customers and workers) do not have mechanisms to pressure platform businesses to offer decent work opportunities. There are some recent international developments around gig worker information sharing and collective organizing such as the growth of platform cooperatives [10]. To further these initiatives and start shifting some of the dramatic informational asymmetries in this area, we believe it is imperative for researchers to produce knowledge that provides a robust understanding of the health and social consequences of this new model of labour relations. Workers’ organization, consumer activism, and government efforts to develop and enforce inclusive labour policies for the well-being of gig workers, all require reliable information to guide policies and programs. Whether platform businesses will engage in some degree of information sharing and transparency remains to be seen.
